# Ratio of fat-free mass to fat mass is associated with physical performance in patients with type 2 diabetes mellitus

**DOI:** 10.3389/fendo.2025.1562870

**Published:** 2025-07-18

**Authors:** Jui-Hsiang Sung, Fu-Shun Ko, Tsung-Hui Wu, Shiow-Chwen Tsai, Chii-Min Hwu, Guan-Yu Su

**Affiliations:** ^1^ Section of Endocrinology and Metabolism, Department of Medicine, Taipei Veterans General Hospital, Taipei, Taiwan; ^2^ Faculty of Medicine, National Yang Ming Chiao Tung University School of Medicine, Taipei, Taiwan; ^3^ Institute of Sports Sciences, University of Taipei, Taipei, Taiwan

**Keywords:** fat-free mass, fat mass, ratio of fat-free mass to fat mass, physical performance, type 2 diabetes mellitus

## Abstract

**Background:**

The ratio of fat-free mass (FFM) to fat mass (FM) is a key indicator of body composition. Evidence suggests that the FFM/FM ratio is more reliable than body mass index in predicting functional outcomes in older adults with prefrailty. Individuals with type 2 diabetes mellitus (T2DM) exhibit higher FM and faster lean mass loss than do those without T2DM. In this study, we determined whether the FFM/FM ratio can predict functional outcomes in patients with T2DM.

**Methods:**

This study enrolled 166 men and 173 women aged ≥50 years who received a T2DM diagnosis at least 1 year before the initiation of this study. Participants were recruited from Taipei Veterans General Hospital, Taiwan, between April 2019 and December 2023. Based on the FFM/FM ratio, patients were stratified into three groups: high, middle, and low tertiles. Body composition was assessed using InBody 3.0. Physical performance was evaluated through the Short Physical Performance Battery and gait speed measurement. The FFM/FM ratio was calculated using established formulas. Stepwise multiple regression was performed to identify the predictors of the FFM/FM ratio in patients stratified by sex.

**Results:**

In both sexes, individuals in the high-tertile group exhibited superior physical performance than did the other groups, as indicated by higher Short Physical Performance Battery scores (*P* < 0.001 for both sexes), better performance in the 30-second chair stand test (*P* < 0.001), faster gait speed in the 6-m walk (*P <*0.001), and shorter completion time in the timed up and go test (*P <*0.001). In men, waist circumference, upper arm circumference, age, logarithmic transformation of alanine transaminase level, and uric acid level emerged as independent predictors of the FFM/FM ratio. In women, waist circumference, upper arm circumference, age, and uric acid level emerged as independent predictors; notably, logarithmic transformation of alanine transaminase level was not included in the regression model.

**Conclusion:**

The present study revealed that a decreased FFM/FM ratio is associated with suboptimal physical performance in patients with T2DM, and this ratio may provide meaningful clinical benefits in targeting body composition in this population.

## 1 Introduction

Fat mass (FM) and fat-free mass (FFM) are commonly used parameters for characterizing body composition. The load–capacity model incorporates both FM and FFM to enhance the prediction of disease risk (1). The relative contributions of FM and FFM to physiological function determine the risks of cardiometabolic diseases, functional disabilities, and sarcopenia (1–4). The ratio of FFM to FM (FFM/FM) has emerged as a meaningful indicator of clinical outcomes across diverse populations. A lower FFM/FM ratio has been associated with increased risks of non-alcoholic fatty liver disease, poor asthma control, and reduced physical performance (5–7). It has also shown predictive value for cardiometabolic risks, with lower ratios more strongly associated with adverse health outcomes (8). Among patients with pancreatic cancer, a reduced FFM/FM ratio has been correlated with worse overall survival (9). Furthermore, the FFM/FM ratio is a key marker for identifying sarcopenic obesity—a condition characterized by metabolic dysfunction and impaired physical function (10). Compared to body mass index (BMI), the FFM/FM ratio provides a more accurate assessment of body composition and has been shown to be a superior predictor of physical performance in prefrail older adults (11).

Type 2 diabetes mellitus (T2DM) is a chronic metabolic disorder characterized by insulin resistance and relative insulin deficiency, leading to hyperglycemia. In addition to its metabolic effects and chronic inflammation, T2DM adversely affects various aspects of physical health, including muscle mass and function. In particular, T2DM alters body composition parameters, such as FM and FFM, thereby compromising physical performance. Adults with T2DM typically gain more FM and lose lean mass more rapidly than do those without T2DM (12).

Although previous studies have analyzed the FFM/FM ratio in older populations that include individuals with T2DM, its role as a reliable predictor of physical performance specifically in patients with T2DM remains uncertain (11). This study aimed to determine whether the FFM/FM ratio can serve as a predictor of functional outcomes in this population. We hypothesized that a lower FFM/FM ratio would be associated with better physical performance in individuals with T2DM. Furthermore, we identified key factors associated with the FFM/FM ratio among these patients.

## 2 Methods

### 2.1 Study design and participants

We enrolled ambulatory patients aged ≥50 years who received a T2DM diagnosis at least 1 year before the commencement of the present study. They were recruited from Taipei Veterans General Hospital, Taiwan, between April 2019 and December 2023. Individuals with an estimated glomerular filtration (eGFR) rate of <15 mL/min/1.73 m^2^ (calculated using the Modification of Diet in Renal Disease Study equation) and those with a history of severe congestive heart failure (New York Heart Association Functional Class III or IV), severe stroke (National Institutes of Health Stroke Scale score > 15), peripheral arterial occlusive disease, or severe muscular disorders were excluded from this study. Patients were ineligible if they had been diagnosed as having foot ulcers or nondiabetic neuropathy 6 months before the present study.

A total of 339 participants (166 men and 173 women) were enrolled in the study, exceeding the calculated minimum sample size of 159 participants required for adequate statistical power. Power calculations were conducted using G*Power 3.1, assuming a medium effect size (f = 0.25), a significance level of α = 0.05, and a power of 0.80. Participants were initially stratified by sex because of known differences in body composition between men and women. Within each sex group, they were further divided into three subgroups based on their FFM/FM ratio: high-tertile, mid-tertile, and low-tertile.

The study protocol was approved by the Ethics Committee of Taipei Veterans General Hospital. Written informed consent was obtained from each patient before their enrollment in this study.

### 2.2 Clinical examination and measurements

Eligible patients underwent anthropometric and blood pressure assessments at 8 am after a 12-h overnight fast. Seated blood pressure was recorded using an automated blood pressure monitor (HEM-7310; Omron Healthcare, Kyoto, Japan). Waist circumference (WC) was measured at the level of the umbilicus to the nearest millimeter. Upper arm circumference (UAC) was measured at the midpoint between the acromion and olecranon processes by using a tape placed on the shoulder blade and ulna, respectively. Calf circumference (CC)was measured at maximal circumference in a seated position, with feet on the floor and the leg positioned at 90°, by using a nonelastic tape.

Blood samples were collected for biochemical analyses. Serum biochemistry was analyzed using commercial assay kits (Roche Diagnostics, Basel, Switzerland) with an automatic blood chemistry analyzer (Roche-Hitachi 7180; Roche Diagnostics). Glycated hemoglobin was detected through capillary electrophoresis.

A bioelectrical impedance analyzer (InBody 3.0; Biospace, Tokyo, Japan) was used to measure various body composition parameters, such as FM, fat percentage, muscle mass, and muscle percentage. FFM was calculated by subtracting body fat weight (body weight × fat percentage) from body weight. The FFM/FM ratio was calculated using a modified version of the Reshma Aziz Merchant formula (7).

### 2.3 Physical performance assessment

Physical performance was evaluated through the Short Physical Performance Battery (SPPB), five times sit-to-stand test, 30-second chair stand test, timed up and go test, and gait speed measurement. The SPPB comprises 3 balance components: the Romberg test, Sharpened Romberg test, and one-leg standing test. Each balance test was conducted twice, once with eyes open and once with eyes closed. The duration (in seconds) for which each participant maintained balance during each test was recorded. The total duration was calculated after the completion of the SPPB. Gait speed was measured by calculating the time taken by each participant to walk a 6-m distance. The average gait speed was calculated from 2 trials.

Handgrip strength of the dominant hand was measured using a Jamar hand dynamometer (Model 5030J1; Patterson Medical, Warrenville, IL, USA). Hand dominance was determined by asking participants which hand they preferred for hammering. The dynamometer was adjusted to either the second or third handle position on the basis of each participant’s comfort level. During the test, each participant stood with the arms relaxed at the sides and elbows fully extended and then exerted the maximum force by squeezing the dynamometer. The highest value from 3 trials was recorded for analysis.

Lower-limb muscle strength of the dominant leg—the leg preferred for kicking a ball—was evaluated using a hand-held dynamometer (microFET2; Hoggan Scientific, Salt Lake City, UT, USA). During a knee extensor strength test, each participant sat with the knees and hips flexed at a 90° angle (short sitting position). Ankle dorsiflexor and plantar flexor strength were measured with the participant in the supine position. A trained examiner instructed the participant to actively apply force against the device while the examiner provided resistance. Three trials were performed for each muscle group, and the highest value was recorded. Muscle strength is expressed in Newtons, calculated as kilograms multiplied by the acceleration due to gravity (9.8 m/s^2^).

### 2.4 Statistical analysis

Continuous variables are presented as mean ± SD or median (interquartile range) values, whereas categorical variables are presented as number (percentage) values. Because of substantial skewness in triglyceride and alanine transaminase (ALT) levels, logarithmic transformation was performed to reduce the skewness before analysis. One-way analysis of variance with the Scheffe *post hoc* test was performed for intergroup comparisons of clinical characteristics, body composition, physical performance, and muscle strength. A general linear model was used to identify the association between the FFM/FM ratio and physical performance; this model was adjusted for handgrip strength or ankle plantor flexor strength.

The associations of various clinical characteristics with the FFM/FM ratio were analyzed by calculating Pearson correlation coefficients. A positive correlation was defined as *r* > 0, while a negative correlation was defined as *r* < 0. An *r* value close to 0 indicates little to no meaningful relationship between the variables. The strength of the correlation was interpreted as follows: 0.00–0.10 (negligible), 0.10–0.39 (weak), 0.40–0.69 (moderate), 0.70–0.89 (strong), and 0.90–1.00 (very strong).

Stepwise multiple regression was performed to identify independent predictors associated with the FFM/FM ratio. In this analysis, the dependent variable was the FFM/FM ratio, whereas the independent variables were age, WC, UAC, CC, systolic blood pressure, diastolic blood pressure, logarithmic transformation of ALT level (logALT) level, eGFR, high-density lipoprotein cholesterol level, logarithmic transformation of triglyceride (logTG) level, uric acid (UA) level, glycated hemoglobin level, and fasting plasma glucose level. A *P* value of <0.05 was indicated statistical significance. All statistical analyses were performed using SPSS (version 25; IBM Corporation, Armonk, NY, USA).

## 3 Results

The study cohort comprised 166 men and 173 women. The mean age of participants was 69.3 ± 8.7 years, and men constituted 49.0% of the cohort. **Table 1** presents the baseline demographic characteristics of the study population. **Table 2** lists the clinical characteristics of participants stratified into high-, mid-, and low-tertile groups based on their FFM/FM ratios.

**Table 1:** Baseline demography of the studied population.

Characteristics	Men	Women	Total
n (%)	166 (49.0)	173 (51.0)	339(100)
Age (years)	69.2 ± 8.8	69.4 ± 8.6	69.3 ± 8.7
BW (kg)	70.8 ± 11.4	61.1 ± 11.2	65.9 ± 12.7
BH (cm)	166.6 ± 6.7	154.7 ± 5.9	160.6 ± 8.7
BMI (kg/m^2^)	25.5 ± 3.4	25.5 ± 4.4	25.5 ± 3.9
WC (cm)	91.0 ± 10.2	88.9 ± 10.7	88.9 ± 10.7
UAC (cm)	29.8 ± 6.9	28.6 ± 4.0	29.2 ± 5.6
CC (cm)	35.8 ± 2.8	33.5 ± 3.5	34.6 ± 3.4
SBP (mmHg)	136.4 ± 17.0	140.9 ± 20.4	138.7 ± 18.9
DBP (mmHg)	79.6 ± 9.7	81.4 ± 9.8	80.5 ± 9.8
FPG (mg/dL)	128.3 ± 33.0	131.2 ± 40.0	129.8 ± 36.7
HbA_1c_ (%)	7.1 ± 1.1	7.3 ± 1.5	7.2 ± 1.3
eGFR	75.3 ± 23.0	78.5 ± 27.5	77.0 ± 25.4
LogALT	1.33 ± 0.20	1.26 ± 0.20	1.30 ± 0.20
HDL (mg/dL)	46.0± 12.3	52.2 ± 13.1	49.1 ± 13.1
LogTG	2.02± 0.20	2.06 ± 0.20	2.04 ± 0.20
UA (mg/dL)	6.0 ± 1.3	5.5 ± 1.3	5.8 ± 1.4

Data are presented as mean ± SD or median (interquartile range) values for continuous variables and number (percentage) values for categorical variables.

ALT, alanine transaminase; LogALT, logarithmic transformation of ALT (in mg/dL); BH, body height; BMI, body mass index; BW, body weight; CC, calf circumference; Cr, creatinine; DBP, diastolic blood pressure; FFM/FM, fat-free mass/fat mass**;** FPG, fasting plasma glucose; HbA1c, glycated hemoglobin; HDL, high-density lipoprotein cholesterol; LDL, low-density lipoprotein cholesterol; SBP, systolic blood pressure; TC, total cholesterol; TG, triglyceride; LogTG, logarithmic transformation of TG (in mg/dL); UA, uric acid; UAC, upper arm circumference; WC, waist circumference.

**Table 2:** Clinical characteristics of the study cohort.

Characteristics	Men (N = 166, 49.0%)				Women (N = 173, 51.0%)			
	Low-tertile group	Mid-tertile group	High-tertile group	*P*	Low-tertile group	Mid-tertile group	High-tertile group	*P*
n (%)	55 (33.1)	55 (33.1)	56 (33.7)		58 (33.5)	58 (33.5)	57 (32.9)	
Age (years)	69.6 ± 9.5	69.8 ± 9.1	68.2 ± 7.8	0.58	68.9 ± 8.8	70.5 ± 7.3	68.7 ± 9.5	0.47
BW (kg)	78.9 ± 12.7	69.6 ± 8.3^a^	64.1 ± 7.1^a,b^	<0.001*	70.6 ± 13.8	58.9 ± 7.4^a^	53.7 ± 7.0^a,b^	<0.001*
BH (cm)	166.0 ± 7.1	166.1 ± 7.2	167.7 ± 5.9	0.33	153.6 ± 6.6	154.3 ± 5.6	156.3 ± 5.2^a^	0.045*
BMI (kg/m^2^)	28.5 ± 3.3	25.1 ± 1.9^a^	22.8 ± 2.1^a,b^	<0.001*	29.7 ± 4.1	24.7 ± 2.2^a^	21.9 ± 2.1^a,b^	<0.001*
WC (cm)	98.7 ± 11.8	90.5 ± 5.5^a^	84.1 ± 6.1^a,b^	<0.001*	97.6 ± 9.8	88.3 ± 8.0^a^	80.5 ± 6.2^a,b^	<0.001*
UAC (cm)	32.3 ± 11.0	28.8 ± 2.3 ^a^	28.3 ± 3.0^a^	0.003*	31.7 ± 4.1	27.9 ± 2.5^a^	26.2 ± 2.8^a,b^	<0.001*
CC (cm)	37.1 ± 3.0	35.8 ± 2.8	34.6 ± 2.1^a^	<0.001*	35.4 ± 3.3	33.2 ± 2.5^a^	31.9 ± 3.6^a^	<0.001*
SBP (mmHg)	136.3 ± 17.1	137.2 ± 16.4	135.8 ± 17.8	0.90	146.1 ± 19.2	136.0 ± 19.3^a^	140.6 ± 21.7	0.03*
DBP (mmHg)	79.3 ± 10.4	79.6 ± 8.4	79.8 ± 10.3	0.96	83.8 ± 8.9	80.8 ± 9.6	79.6 ± 10.5	0.06
FPG (mg/dL)	130.0 ± 37.0	126.5 ± 28.7	128.3 ± 33.3	0.86	131.6 ± 36.8	136.5 ± 47.1	125.5 ± 34.9	0.34
HbA_1c_ (%)	7.0 ± 0.9	7.1 ± 1.2	7.2 ± 1.1	0.81	7.3 ± 1.2	7.4 ± 1.5	7.1 ± 1.7	0.54
eGFR	71.6 ± 24.6	76.7 ± 22.9	77.6 ± 21.3	0.33	76.3 ± 28.2	74.0 ± 25.1	85.4 ± 28.1	0.06
LogALT	1.35 ± 0.24	1.36 ± 0.20	1.28 ± 0.14	0.90	1.27 ± 0.21	1.29 ± 0.20	1.22 ± 0.18	0.20
HDL (mg/dL)	41.4 ± 11.5	46.0 ± 12.2	50.0 ± 11.9	0.10	49.8 ± 10.8	51.3 ± 14.5	55.4 ± 13.2	0.27
LogTG	2.08 ± 0.22	2.02 ± 0.17	1.96 ± 0.19^a^	0.01*	2.08 ± 0.16	2.07 ± 0.21	2.01 ± 0.21	0.10
UA (mg/dL)	6.1 ± 1.3	6.0 ± 1.3	5.9 ± 1.3	0.37	5.7 ± 1.4	5.6 ± 1.4	5.1 ± 1.1	0.05

Data are presented as mean ± SD or median (interquartile range) values for continuous variables and number (percentage) values for categorical variables. One-way analysis of variance was performed for intergroup comparisons of variables.

ALT, alanine transaminase; LogALT, logarithmic transformation of ALT (in mg/dL); BH, body height; BMI, body mass index; BW, body weight; CC, calf circumference; Cr, creatinine; DBP, diastolic blood pressure; FFM/FM, fat-free mass/fat mass**;** FPG, fasting plasma glucose; HbA1c, glycated hemoglobin; HDL, high-density lipoprotein cholesterol; LDL, low-density lipoprotein cholesterol; SBP, systolic blood pressure; TC, total cholesterol; TG, triglyceride; LogTG, logarithmic transformation of TG (in mg/dL); UA, uric acid; UAC, upper arm circumference; WC, waist circumference.

*P < 0.05.

a *P* < 0.05, compared with the low-tertile group.

b *P* < 0.05, compared with the mid-tertile group.

Among men, the low-tertile group had significantly larger WC, UAC, and CC than did the mid-tertile and high-tertile groups (WC: 98.7 ± 11.8 vs 90.5 ± 5.5 vs 84.1 ± 6.1 cm [*P* < 0.001]; UAC: 32.3 ± 11.0 vs 28.8 ± 2.3 vs 28.3 ± 3.0 cm [*P* = 0.003]; CC: 37.1 ± 3.0 vs 35.8 ± 2.8 vs 34.6 ± 2.1 cm [*P* < 0.001]). Furthermore, the level of LogTG was significantly higher in the low-tertile group than in the mid-tertile and high-tertile groups (2.08 ± 0.22, 2.02 ± 0.17, and 1.96 ± 0.19, respectively, *P* = 0.01). Similarly, among women, the low-tertile group had significantly larger WC, UAC, and CC than did the mid-tertile and high-tertile groups (WC: 97.6 ± 9.8 vs 88.3 ± 8.0 vs 80.5 ± 6.2 cm [*P* < 0.001]; UAC: 31.7 ± 4.1 vs 27.9 ± 2.5 vs 26.2 ± 2.8 cm [*P* < 0.001]; CC: 35.4 ± 3.3 vs 33.2 ± 2.5 vs 31.9 ± 3.6 cm [*P* < 0.001]). However, no significant intergroup difference was observed in LogTG level (*P* = 0.10).

**Figure 1:**
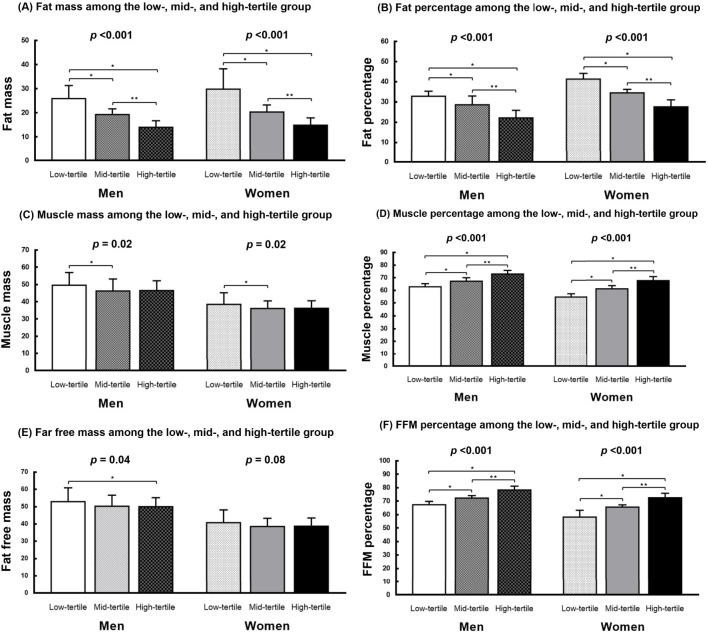
Body composition of the study cohort. **(A, B)** The FFM/FM ratio showed a negative correlation with both FM and FM percentage in men and women. **(C, E)** Significant differences between groups were observed in both muscle mass and FFM among men, whereas in women, significant intergroup differences were observed only in muscle mass. **(D, F)** The FFM/FM ratio showed a positive association with muscle mass percentage and FFM percentage in both men and women. FM, fat mass; FFM, fat-free mass; *P < 0.05, compared with the low-tertile group. **P <0.05, compared with the mid-tertile group.

**Figure 1** presents the body composition of the 3 groups. In both men and women, the FFM/FM ratio was negatively correlated with FM, indicating that a higher FFM/FM ratio was associated with a lower FM (men: *P* < 0.001; women: *P* < 0.001; **Figure 1A**). Similarly, the FFM/FM ratio was negatively associated with FM percentage (men: *P* < 0.001; women: *P* < 0.001; **Figure 1B**). However, the FFM/FM ratio was positively associated muscle mass percentage (men: *P* < 0.001; women: *P* < 0.001; **Figure 1D**) and FFM percentage (men: *P* < 0.001; women: *P* < 0.001; **Figure 1F**). In men, significant intergroup differences were noted in both muscle mass (*P* = .02) and FFM (*P* = 0.04). By contrast, in women, significant intergroup differences were noted in only muscle mass (*P* = 0.02; **Figure 1C**), but not in FFM (**Figure 1E**).

In both men and women, the high-tertile group exhibited better physical performance than did the low- and mid-tertile groups (**Supplementary Table 1**). In women, handgrip strength and muscle strength of the ankle plantar flexor were significantly higher in the high-tertile group than in the mid-tertile and low-tertile groups (handgrip strength: 229.4 ± 66.9 vs 217.0 ± 48.3 vs 198.7 ± 46.3 [*P* = 0.01]; ankle plantor flexor strength: 434.8 ± 94.6 vs 394.4 ± 84.4 vs 400.1 ± 103.2 [*P* = 0.049]; **Supplementary Table 2**). By contrast, in men, no significant intergroup differences were noted in handgrip strength or ankle plantar flexor strength (*P* = 0.17 and *P* = 0.37, respectively; **Supplementary Table 2**).

In both men and women, the general linear model adjusted for handgrip strength or ankle plantor flexor strength identified the FFM/FM ratio as an independent predictor of physical performance (**Tables 3**, **4**). In both sex groups, the high-tertile group exhibited superior physical performance than did the other groups. The high-tertile group achieved higher SPPB scores (*P* < 0.001 for both sexes), completed the 5 times sit-to-stand test in less time (*P* < 0.001 for both sexes), performed better in the 30-second chair stand test (*P* < 0.001 for both sexes), attained faster gait speeds in the 6-m walk (*P <*0.001 for both sexes), and completed the timed up and go test more rapidly (*P* < 0.001 for both sexes) than did the mid-tertile and low-tertile groups.

**Table 3:** Association of physical performance with the FFM/FM ratio in men.

Physical performance	Model 1				Model 2			
	Low-tertile group	Mid-tertile group	High-tertile group	*P*	Low-tertile group	Mid-tertile group	High-tertile group	*P*
*SPPB*	109.8 (103.0-116.5)	115.1 (108.3-121.9)	118.0 (111.2-124.7)	<0.001*	108.1 (101.9-114.3)	115.7 (109.5-121.9)	119.0 (112.9-125.1)	0.001
FTSST	9.83 (9.15-10.52)	8.63 (7.94-9.31)	8.87 (8.19-9.55)	<0.001*	9.96 (9.28-10.64)	8.62 (7.94-9.30)	8.75 (8.08-9.42)	<0.001*
30CST	17.3 (15.9-18.6)	19.3 (18.0-20.7)	19.8 (18.4-21.1)	<0.001*	17.0 (15.7-18.2)	19.4 (18.2-20.7)	20.0 (18.7-21.2)	<0.001*
Speed of 6M walk (m/s)	1.07 (1.01-1.13)	1.15 (1.09-1.21)	1.18 (1.12-1.24)	<0.001*	1.05 (1.00-1.11)	1.15 (1.10-1.21)	1.19 (1.13-1.24)	<0.001*
TUG test (sec)	8.27 (7.78-8.75)	7.59 (7.11-8.08)	7.28 (6.80-7.76]	<0.001*	8.40 (7.96-8.84)	7.55 (7.11-7.99)	7.19 (6.76-7.62)	<0.001*

Data are presented as mean and 95% CI values. A general linear model was used to identify the association between the FFM/FM ratio and physical performance.

FFM, fat-free mass; FM, fat mass; SPPB, Short Physical Performance Battery; 6M walk, 6-m walk; FTSST, five times sit-to-stand test; 30CST, 30-second chair stand test; TUG, timed up and go test.

Model 1 was adjusted for the handgrip strength.

Model 2 was adjusted ankle plantor flexor strength.

*P < 0.05.

**Table 4:** Association of physical performance with the FFM/FM ratio in women.

Physical performance	Model 1				Model 2			
	Low-tertile group	Mid-tertile group	High-tertile group	*P*	Low-tertile group	Mid-tertile group	High-tertile group	*P*
*SPPB*	101.8 (94.7-108.9)	108.6 (101.6-115.6)	114.1 (106.9-115.6)	<0.001*	100.8 (93.9-107.6)	110.3 (103.4-117.1)	113.4 (106.5-120.4)	<0.001*
FTSST	10.64 (9.73-11.55)	9.89 (8.99-10.79)	9.33 (8.42-10.25)	<0.001*	10.98 (10.05-11.91)	9.63 (8.69-10.56)	9.25 (8.30-10.20)	<0.001*
30CST	16.3 (15.0-17.7)	17.2 (15.8-18.5)	18.4 (17.0-19.7)	<0.001*	15.8 (14.5-17.2)	17.5 (16.1-18.9)	18.5 (17.1-19.9)	<0.001*
Speed of 6M walk (m/s)	1.01 (0.96-1.07)	1.05 (0.98-1.09)	1.09 (1.04-1.15)	<0.001*	0.99 (0.93-1.04)	1.05 (1.00-1.11)	1.10 (1.04-1.15)	<0.001*
TUG test (sec)	9.03 (8.41-9.65)	8.40 (7.79-9.01)	8.18 (7.55-8.80)	<0.001*	9.26 (8.65-9.87)	8.17 (7.56-8.78)	8.18 (7.56-8.80)	<0.001*

Data are presented as mean and 95% CI values. A general linear model was used to identify the association between the FFM/FM ratio and physical performance.

FFM, fat-free mass; FM, fat mass; SPPB, Short Physical Performance Battery; 6M walk, 6-m walk; FTSST, five times sit-to-stand test; 30CST, 30-second chair stand test; TUG, timed up and go test.

Model 1 was adjusted for the handgrip strength.

Model 2 was adjusted for ankle plantor flexor strength.

*P < 0.05.

The results of Pearson correlation analyses are summarized in **Tables 5**, **6**. In men, the FFM/FM ratio was significantly associated with WC, UAC, CC, LogALT level, eGFR, HDL level, LogTG level, and UA level. Similarly, in women, the FFM/FM ratio was significantly associated with WC, UAC, CC, HDL level, LogTG level, and UA level. **Table 7** presents key factors influencing the FFM/FM ratio. In men, WC emerged as the primary predictor of the FFM/FM ratio in the first step, followed by UAC (second step), age (third step), LogALT level (fourth step), and UA level (fifth step). In women, WC emerged as the primary predictor of the FFM/FM ratio in the first step, followed by UAC (second step), age (third step), and UA level (fourth step). All of these factors independently predicted the variance in the FFM/FM ratio.

**Table 5:** Associations of various clinical characteristics with the FFM/FM ratio in men.

Variables	FFM/FM	Age	WC	UAC	CC	SBP	DBP	FPG	HbA1c	LogALT	eGFR	HDL	LogTG
Age	-0.131												
WC	-0.621^**^	-0.035											
UAC	-0.248^**^	-0.217^**^	-0.154^*^										
CC	-0.427^**^	-0.341^**^	0.389^**^	0.401^**^									
SBP	-0.102	0.242^**^	0.034	0.060	-0.026								
DBP	-0.040	-0.321^**^	0.095	0.201^**^	0.229^**^	0.338^**^							
FPG	-0.028	-0.040	0.047	0.005	0.119	0.037	0.061						
HbA_1c_	0.026	-0.064	0.053	0.008	0.127	-0.133	0.046	0.444^**^					
LogALT	-0.165^*^	-0.092	0.062	0.028	0.096	-0.035	0.006	0.179^*^	0.176^*^				
eGFR	0.199^*^	-0.276^**^	-0.085	-0.013	-0.047	-0.198^*^	0.044	-0.047	-0.017	0.174^*^			
HDL	-0.275^**^	-0.117	0.182^*^	0.246^**^	0.108	0.110	0.259^**^	0.176^*^	0.094	0.130	-0.123		
LogTG	0.295^**^	-0.007	-0.297^**^	-0.061	-0.105	0.209^**^	0.027	-0.003	-0.148	-0.111	0.143	-0.272^**^	
UA	-0.177^*^	0-.005	0.075	-0.012	0.089	0.165^*^	0.116	-0.003	0.039	0.010	-0.291^**^	0.201^**^	-0.120

Abbreviations as in **Table 1**.

*P < 0.05 and **P < 0.01 (Pearson correlation analysis).

**Table 6:** Associations of various clinical characteristics with the FFM/FM ratio in women.

Variables	FFM/FM	Age	WC	UAC	CC	SBP	DBP	FPG	HbA1c	LogALT	eGFR	HDL	LogTG
Age	-0.024												
WC	-0.660^**^	-0.089											
UAC	-0.598^**^	-0.244^**^	0.722^**^										
CC	-0.416^**^	-0.308^**^	0.579^**^	0.697^**^									
SBP	-0.106	0.254^**^	0.053	0.050	0.026								
DBP	-0.198^**^	-0.038	0.135	0.142	0.113	0.596^**^							
FPG	-0.044	-0.115	0.048	0.021	-0.024	0.046	0.090						
HbA_1c_	0.014	-0.172^*^	0.126	0.095	0.069	0.083	-0.001	0.596^**^					
LogALT	-0.101	-0.097	0.103	0.147	0.088	0.009	0.102	0.258^**^	0.154^*^				
eGFR	0.117	-0.299^**^	-0.106	-0.041	-0.063	-0.158^*^	0.009	0.000	-0.095	0.086			
HDL	0.191^*^	0.046	-0.260^**^	-0.171^*^	-0.044	-0.058	-0.164^*^	-0.200^**^	-0.175^*^	-0.152^*^	0.144		
LogTG	-0.220^**^	-0.015	0.210^**^	0.165^*^	0.077	0.062	0.116	0.179^*^	0.201^**^	0.207^**^	-0.199^**^	-0.520^**^	
UA	-0.206^**^	0.008	0.155^*^	0.086	0.079	0.075	0.035	0.042	0.011	0.015	-0.514^**^	-0.154^*^	0.221^**^

Abbreviations as in **Table 1**.

*P < 0.05 and **P < 0.01 (Pearson correlation analysis).

**Table 7:** Key factors influencing the FFM/FM ratio (N = 339).

Model	Gender	Variables entered	Estimate	SE	*P*	*R ^2^*
1	Men	Waist circumference	-3.0 x 10^-2^	0.003	<0.001*	0.38
2	Men	Waist circumference	-3.3 x 10^-2^	0.003	<0.001*	0.50
		Upper arm circumference	-2.6 x 10^-2^	0.004	<0.001*	
3	Men	Waist circumference	-3.4 x 10^-2^	0.003	<0.001*	0.56
		Upper arm circumference	-3.0 x 10^-2^	0.004	<0.001*	
		Age	-1.4 x 10^-2^	0.003	<0.001*	
4	Men	Waist circumference	-3.3 x 10^-2^	0.003	<0.001*	0.57
		Upper arm circumference	-2.9 x 10^-2^	0.004	<0.001*	
		Age	-1.4 x 10^-2^	0.003	<0.001*	
		LogALT	3.4 x 10^-1^	0.130	0.01*	
5	Men	Waist circumference	-3.3 x 10^-2^	0.003	<0.001*	0.59
		Upper arm circumference	-2.9 x 10^-2^	0.004	<0.001*	
		Age	-1.4 x 10^-2^	0.003	<0.001*	
		LogALT	-3.9 x 10^-2^	0.128	0.01*	
		UA	-4.9 x 10^-2^	0.019	0.01*	
1	Women	Waist circumference	-3.9 x 10^-2^	0.003	<0.001*	0.42
2	Women	Waist circumference	-2.8 x 10^-2^	0.005	<0.001*	0.46
		Upper arm circumference	-4.1 x 10^-2^	0.013	<0.001*	
3	Women	Waist circumference	-2.7 x 10^-2^	0.005	<0.001*	0.48
		Upper arm circumference	-4.9 x 10^-2^	0.013	<0.001*	
		Age	-1.0 x 10^-2^	0.004	0.02*	
4	Women	Waist circumference	-2.5 x 10^-2^	0.005	<0.001*	0.49
		Upper arm circumference	-5.0 x 10^-2^	0.013	<0.001*	
		Age	-1.0 x 10^-2^	0.004	0.02*	
		UA	-5.2 x 10^-2^	0.026	0.046*	

Abbreviations as in **Table 1**.

Dependent variable: the FFM/FM ratio.

Independent variables: age, WC, UAC, CC, SBP, DBP, LogALT level, eGFR, HDL level, LogTG level, UA level, HbA1c level, and FPG level. *p < 0.05.

## 4 Discussion

The present study revealed that body composition, particularly an elevated FFM/FM ratio, was associated with enhanced physical performance in patients with T2DM. In both men and women, a higher FFM/FM ratio was associated with lower body weight and BMI and smaller WC, UAC, and CC. In men, WC was the primary predictor of the FFM/FM ratio, followed by UAC, age, LogALT level, and UA level. Similarly, in women, WC was the primary predictor of the FFM/FM ratio, followed by UAC, age, and UA level.

T2DM substantially affects physical performance in individuals ≥50 years by reducing muscle strength, impairing balance, and increasing the fear of falling (13–19). Older adults with T2DM have reduced handgrip strength, which is correlated with slow gait speed and chair stand speed, indicating compromised functional abilities (13). Furthermore, balance-related problems are highly prevalent among patients with T2DM, increasing the risk of falls and the fear of falling in this population; the fear of falling deters these individuals from engaging in physical activity and reduces their quality of life (15, 16).

In the present study, physical performance in patients with T2DM was evaluated through five key tests. These included the SPPB (lower scores indicate reduced mobility), the five times sit-to-stand, and the 30-second chair stand tests, which measure lower limb strength and endurance (19–21). Gait speed was assessed by the 6-m walk test, as recommended by the Asian Working Group for Sarcopenia (22). The timed up and go test was used to evaluate fall risk and mobility and has been linked to mortality prediction in older adults (23). A general linear model adjusted for muscle strength showed that a lower FFM/FM ratio, in both men and women, was associated with poorer physical performance.

A lower FFM/FM ratio typically reflects a reduction in fat-free mass, an increase in fat mass, or both. FFM encompasses skeletal muscle, bone, body water, and internal organs, with skeletal muscle playing a critical role in maintaining strength, balance, endurance, and mobility. In individuals with T2DM, sarcopenia—the progressive loss of skeletal muscle mass—is frequently observed, particularly with advancing age and worsening insulin resistance. This decline in muscle mass, which indicated a decrease of muscle strength, significantly impairs mobility and increases the risk of disability (24).

Conversely, excess fat mass, particularly visceral adiposity, exerts multiple harmful effects on muscle health. Findings from the Health, Aging, and Body Composition Study demonstrated that greater adiposity is associated with reduced muscle quality and predicts an accelerated decline in lean mass (25). Visceral fat may contribute to both inter- and intramuscular fat infiltration, which can impair mitochondrial function, elevate the production of reactive oxygen species, and trigger the secretion of pro-inflammatory myokines (26). Moreover, visceral adipose tissue releases cytokines such as tumor necrosis factor-alpha (TNF-α) and interleukin-6 (IL-6), which further promote muscle catabolism by suppressing protein synthesis and enhancing proteolysis (27). Increased fat mass also exacerbates insulin resistance, compromising glucose and amino acid uptake in skeletal muscle and thereby impairing muscle repair and regeneration (28). These mechanisms may explain why the FFM/FM ratio serves as a potential indicator of functional health.

In both men and women with T2DM, WC, UAC, and age emerged as significant predictors of the FFM/FM ratio. WC is a strong predictor of visceral adiposity and is associated with insulin resistance and cardiovascular risk in patients with T2DM (29, 30). A higher WC indicates central obesity with increased FM, which is correlated with a lower FFM/FM ratio. UAC serves as a measure of skeletal muscle mass and strength, both of which are crucial indicators of physical performance and metabolic health. The Asian Working Group for Sarcopenia recommends UAC as a screening tool for sarcopenia (22, 31, 32). In our study, a smaller UAC was associated with a higher FFM/FM ratio, indicating relatively greater muscle mass and better physical performance (32). Aging is associated with changes in body composition—for example, the loss of muscle mass and an increase in FM, which reduce the FFM/FM ratio (11, 33).

To the best of our knowledge, this study is the first to investigate whether the FFM/FM ratio could predict physical performance in Asian patients with T2DM. A relevant study reported that age, sex, and BMI influenced the FM/FFM ratio in older adults (7). However, in the present study, BMI was not identified as a significant predictor of the FFM/FM ratio in patients with T2DM. This finding may be attributable to the moderate correlation of BMI with T2DM and other chronic diseases (34). In addition, although BMI is commonly used to define obesity, it does not differentiate between FM and FFM (35). A study on BMI and WC revealed that a higher BMI was associated with a better functional and cognitive status, particularly in men (36). These findings explain why BMI exerted no significant effect on the FFM/FM ratio in our study.

This study has several limitations. First, the cross-sectional design of our study precludes the evaluation of longitudinal changes in physical function relative to changes in the FFM/FM ratio. Second, as a single-center study with a relatively small sample size, the generalizability of our findings to the broader population may be limited. Third, FM and FFM were estimated using bioelectrical impedance analysis (BIA), which, though endorsed by the Asian Working Group for Sarcopenia (2019) and strongly correlated with dual-energy X-ray absorptiometry (DEXA), may still introduce potential measurement imprecision (22, 37). Fourth, detailed information on diabetes onset and duration, daily physical activity levels, nutritional assessment parameters, and pharmacological treatments for glycemic control were not quantitatively recorded. Their absence from the statistical model may have influenced the observed relationship between body composition and functional capacity, thereby limiting the overall accuracy and reliability of the study’s findings. Additionally, our regression model explained only approximately 59% and 49% of the variance in the FFM/FM ratio for men and women, respectively. Finally, our study lacks a normoglycemic control cohort. Without this comparative group, it remains unclear whether the observed association between the FFM/FM ratio and physical performance represents a diabetes-specific phenomenon or a general physiological relationship.

## 5 Conclusion

Our study revealed a significant association between the FFM/FM ratio and physical performance in patients with T2DM, with lower ratios linked to poorer performance in both sexes. WC, UAC, and age were identified as significant predictors of the FFM/FM ratio in both men and women. Interventions targeting the improvement of body composition—such as personalized exercise programs, dietary modifications, and lifestyle changes—could provide meaningful clinical benefits. To further advance this research, future research should adopt longitudinal designs, incorporate a wider range of clinical and metabolic variables, and utilize advanced imaging techniques to enhance measurement precision and strengthen the evidence supporting the relationship between body composition and functional health.

## Data Availability

The original contributions presented in the study are included in the article/**Supplementary Material**. Further inquiries can be directed to the corresponding authors.
