# Supplementary material for: Ratio of fat-free mass to fat mass is associated with physical performance in patients with type 2 diabetes mellitus
Source: Front Endocrinol (Lausanne). 2025 Jul 18;16:1562870. doi: 10.3389/fendo.2025.1562870 (PMC12313479; doi:10.3389/fendo.2025.1562870)
Supplement: Supplementary file 1 [file Table1.docx]

**Supplementary Table 1.** Physical Performance of the Study Cohort

| **Physical performance** | **Men** | | | | **Women** | | | |
| --- | --- | --- | --- | --- | --- | --- | --- | --- |
|  | **Low-tertile group** | **Mid-tertile group** | **High-tertile group** | ***P*** | **Low-tertile group** | **Mid-tertile group** | **High-tertile group** | ***P*** |
| n (%) | 55 (33.1) | 55 (33.1) | 56 (33.7) |  | 58 (33.5) | 58 (33.5) | 57 (32.9) |  |
| SPPB | 109.7 ± 28.7 | 113.8 ± 26.0 | 119.0 ± 25.0 | 0.16 | 99.9 ± 28.0 | 108.9 ± 25.9 | 115.8 ± 29.2^a^ | 0.01* |
| FTSST | 9.84 ± 3.12 | 8.77 ± 2.48 | 8.72 ± 2.53 | 0.05 | 11.11 ± 4.37 | 9.83 ± 4.01 | 8.91 ± 2.80^a^ | 0.01* |
| 30CST | 17.3 ± 5.2 | 19.1 ± 5.0^a^ | 20.0 ± 5.9^a^ | 0.03* | 15.7 ± 5.4 | 17.3 ± 5.3 | 19.0 ± 6.0^a^ | 0.01* |
| Speed of 6M walk (m/s) | 1.07 ± 0.22 | 1.14 ± 0.28 | 1.19 ± 0.19^a^ | 0.02* | 0.98 ± 0.22 | 1.04 ± 0.24 | 1.12 ± 0.23^a^ | 0.01* |
| TUG test (sec) | 8.27 ± 2.25 | 7.70 ± 2.07 | 7.16 ± 1.42^a^ | 0.01* | 9.37 ± 2.86 | 8.36 ± 2.01 | 7.87 ± 2.88^a^ | 0.01* |

Data are presented as mean ± SD or median (interquartile range) values for continuous variables and number (percentage) values for noncontinuous variables. One-way analysis of variance was performed for intergroup comparisons of variables.

SPPB, Short Physical Performance Battery; 6M walk, 6-m walk; FTSST, five times sit-to-stand test; 30CST, 30-second chair stand test; TUG, timed up and go test.

**P* < 0.05.

^a^*P* < 0.05, compared with the low-tertile group.

^b^*P* < 0.05, compared with the mid-tertile group.

**Supplementary Table 2.** Muscle Strength (kg·m/s^2^) of the Study Cohort

| **Muscle strength** | **Men** | | | | **Women** | | | |
| --- | --- | --- | --- | --- | --- | --- | --- | --- |
|  | **Low-tertile group** | **Mid-tertile group** | **High-tertile group** | ***P*** | **Low-tertile group** | **Mid-tertile group** | **High-tertile group** | ***P*** |
| n (%) | 55 (33.1) | 55 (33.1) | 56 (33.7) |  | 58 (33.5) | 58 (33.5) | 57 (32.9) |  |
| Hand grip (D) | 317.2 ± 59.2 | 307.7 ± 69.6 | 328.3 ± 65.8 | 0.25 | 198.7 ± 46.3 | 217.0 ± 48.3 | 229.4 ± 66.9^a^ | 0.01* |
| Knee extensor | 373.3 ± 66.3 | 347.4 ± 81.9 | 368.3 ± 61.8 | 0.13 | 299.6 ± 76.1 | 301.1 ± 65.4 | 309.1 ± 64.2 | 0.73 |
| Ankle dorsiflexor | 238.3 ± 45.0 | 228.7 ± 45.9 | 234.5 ± 45.9 | 0.53 | 181.9 ± 48.3 | 177.8 ± 37.6 | 177.6 ± 35.7 | 0.81 |
| Ankle plantor | 495.8 ± 113.6 | 465.9 ± 124.4 | 485.0 ± 95.1 | 0.37 | 400.1 ± 103.2 | 394.4 ± 84.4 | 434.8 ± 94.6^b^ | 0.049* |

Data are presented as mean ± SD or median (interquartile range) values for continuous variables and number (percentage) values for non-continuous variables. One-way analysis of variance was performed for intergroup comparisons variables.

D indicates dominant.

**P* < 0.05.

^a^*P* < 0.05 compared with the low-tertile group.

^b^*P* < 0.05, compared with the mid-tertile group.
